# Ex vivo tumor dissection followed by kidney autotransplantation in bilateral wilms tumor

**DOI:** 10.3389/fped.2023.1120797

**Published:** 2023-02-03

**Authors:** Zhihai Zhong, Hong Jiang, Huadong Chen, Chenglin Wu, Yuanqi Wang, Zhichong Zhang, Jun Li, Juncheng Liu

**Affiliations:** ^1^ Department of Pediatric Surgery, First Affiliated Hospital, Sun Yat-sen University, Guangzhou, China; ^2^ Department of Organ Transplantation, First Affiliated Hospital, Sun Yat-sen University, Guangzhou, China

**Keywords:** bilateral wilms tumor, ex vivo tumor dissection, kidney autotransplantation, postoperative complication, tumor-free survival

## Abstract

**Introduction:**

Successful management of bilateral Wilm's tumor (BWT) involves a radical resection while preserving enough normal kidney tissue. Nephron-sparing surgery often results in an R1/R2 resection with a high recurrence rate in children with huge or multiple tumors, or tumors proximity to the renal hilum. In contrast, kidney autotransplantation can completely resect the tumor while maintaining homeostasis and preserving the patient's healthy kidney tissues.

**Methods:**

We summarized the clinical data of 8 synchronous BWT patients who underwent kidney autotransplantation at the First Affiliated Hospital of Sun Yat-sen University from 2018 to 2020. *Ex vivo* tumor resection and kidney autotransplantions were performed on 11 kidneys. The baseline characteristics, perioperative management, and survival status were reported.

**Results:**

Nephron-sparing surgeries were performed on 5 kidneys *in vivo*. Among all the 8 patients, six of them (75%) received staged operation and the other 2 patients (25%) received single-stage operation. No residual tumors were found on the postoperative imaging in all the 8 patients. In total, 6 (75%) patients occurred complications after the autotransplantation, among which, 2 (33.3%) patients had complication of Clavien-Dindo grade IIIa, and 4 (66.7%) patients had complication of grade < 3. During the 38 months of follow-up, 87.5% (7/8) of patients were tumor-free survival with normal renal function. One patient died from renal failure without tumor recurrence.

**Discussion:**

Therefore, our study indicated that autologous kidney transplantation can be an option for patients with complex BWT if the hospital's surgical technique and perioperative management conditions are feasible.

## Introduction

Wilms tumor (WT) is the most common malignant pediatric renal tumor, with an incidence rate of about 1 in 10,000 (1). Among them, synchronous bilateral Wilms tumor (BWT) accounts for about 5% (2). The challenge in the management of BWT lies in ensuring a radical resection while preserving enough normal kidney tissue to ensure the child's normal growth and development. Recently, the long-term survival rate of children with BWT has reached more than 80%. This is mostly due to the advancement of neoadjuvant chemotherapy and surgical techniques (3, 4). However, around 10% of children with BWT have too little residual renal tissue after neoadjuvant therapy and will therefore develop end-stage renal disease, affecting their postoperative quality of life, growth, and development (5, 6).

Currently, bilateral nephron-sparing surgery (NSS) is commonly used for the surgical management of BWT. However, in complex cases (such as children with huge tumors, intraoperative insufficient visual field exposure, or massive blood loss that interferes with the operative field) we can only perform an R1/R2 resection, resulting in a high recurrence rate (7). The 5-years recurrence or mortality rate for these patients is reported to be about 40% (7–9). Hence, it's urgent to achieve both complete resection of tumor and adequate residual kidney tissue for normal function, growth, and development of BWT patients.

Renal autotransplantation was first reported by Professor James Hardy in the United States (10). It is often used to treat patients with complex kidney tumors, ureteral diseases, or abnormal renal arteries caused by arteritis and vascular malformation. The surgical procedure is performed as follows: the kidney is surgically removed and cooled down on ice. Surgical management (such as resection of the tumor, and correction of vascular malformation) is performed on the cooled-down kidney *ex vivo*. The organ is then retransplanted. Autologous kidney transplantation can completely resect the tumor while maintaining homeostasis and preserving the patient's normal kidney tissues. Previous literatures had described the technique in BWT two decades before with small sample size (11, 12). The use of advanced surgical techniques, instruments, equipment, and multidisciplinary cooperation has improved current surgical methods. We believe that neoadjuvant chemotherapy, combined with ex vivo excision of renal tumors and kidney autotransplantation can provide an alternative method for BWT patients. This method is suitable for patients with huge renal tumors, multiple lesions in the kidney, or with an invasion of the renal pelvis. Herein, we summarized the clinical data of 8 patients with synchronous BWT, who underwent kidney autotransplantation with good prognosis and safety at our center.

## Materials and methods

### Patients

A retrospective analysis of the clinical data of Wilms tumor patients admitted to the First Affiliated Hospital of Sun Yat-sen University from 2018 to 2020 was performed. Patients with synchronous BWT who accepted unilateral/bilateral kidney autotransplantation as part of the surgical management were enrolled. Patient demographics (age, sex, size and stage of bilateral tumors, chemotherapeutic regimen, combined diseases) and perioperative data (operation method, use of intraoperative blood transfusion, length of operation, postoperative complications, pathological diagnosis) were retrospectively collected.

All the patients and their parents gave informed consent. This study was approved by the Clinical Research and Experimental Animal Ethics Committee of the First Affiliated Hospital of Sun Yat-sen University [Approval number: (2021)129].

### Treatment

According to the 2016 Wilms tumor guidelines of the International Society of Pediatric Oncology (SIOP) (13), children with bilateral Wilms tumors first received neoadjuvant chemotherapy. Preoperative chemotherapy drugs included Actinomycin D combined with Vincristine and Epirubicin. Surgical assessment using imaging techniques was first performed after 2 cycles of chemotherapy, and thereafter after every 2 cycles. In non-resectable cases, the number of preoperative chemotherapy courses was increased.

If deemed resectable, one of the 3 following operation methods was chosen: 1. Radical nephrectomy was performed for huge tumors when the kidney could not be preserved at all. 2. NSS surgery was performed for small tumors that could be removed *in vivo* completely. 3. Renal tumor ex vivo resection and autologous kidney in-situ transplantation was selected for huge or multiple tumors adjacent to the renal hilum; or located in the deep middle of kidney, or proximal to renal vessels.

Intraoperatively, the patient was placed in a supine position, the lumbar region was elevated, and a transverse incision was made on the upper abdomen. Intraoperatively, the renal hilar vessels and ureters were freed, and sufficient lengths of the ureters and renal hilar vessels were reserved. The proximal renal artery and vein were clamped with blood vessel clamps. The distal ureter was also handled, and the kidney (including the tumor) was removed. While removing the renal tumor *in vitro* and processing the renal pelvis and blood vessels, another group of surgeons removed the retroperitoneal lymph nodes.

**Figure 1:**
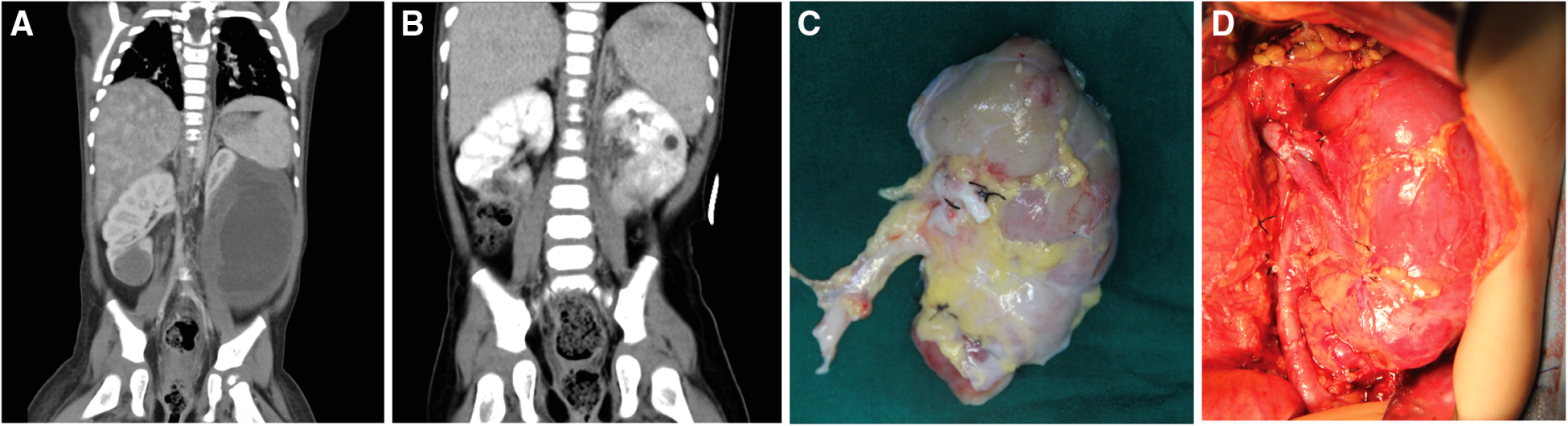
Preoperative images of patients and photos of the surgical area. (**A**) The preoperative enhanced CT image of patient No. 8 showed a tumor size of 83 × 62 × 116 mm in the lower pole of the left kidney and a tumor size of 28 × 23 × 26 mm in the lower pole of the right kidney; (**B**) The post-operative enhanced CT image of patient No. 8, the renal tumor has been completely resected; (**C**) The left kidney was completely stripped of the tumor *in vitro*, and the residual kidney was sutured; (**D**) The left kidney was autotransplanted.

The isolated kidney was immediately placed in sterile ice pellets to lower the kidney's temperature. Within 2 min, 4 °C hypertonic citrate adenine solution (HCA) cryopreservation solution was simultaneously injected. The preservation solution was perfused in the kidney to completely replace the residual blood. This perfusion was continued during the whole process of removing tumors and kidney repairing *in vitro* to keep the kidney in a bloodless state. After incising the renal capsule, tumors were peeled from the normal tissue carefully with complete tumor capsule. Hemostasis and ligation were given to blood vessels at the cutting edge, and renal arteries and veins were trimmed for anastomosis. Then, we repaired the damaged renal system, and sutured the renal capsule. The excised and processed tumor-free kidney was orthotopically implanted into the body (Figure 1). The renal artery and vein were sutured end-to-end. The ureter was also anastomosed end-to-end, with a built-in double J tube. Depending on the surgeon's experience, a ureteral stent can be placed during the operation for drainage, to reduce anastomotic pressure. A perinephric drainage tube can also be placed at the same time to drain the leaked urine.

### Postoperative management and follow-up

Postoperative renal function and blood supply were assessed by monitoring the hourly urine output, and the occurrence of any hematuria. An ultrasound examination assessing the blood supply of the kidneys was performed on postoperative day 1. The urinary catheter was routinely indwelled for 3–5 days and was removed after the urine volume and color became normal. During the perioperative period, the patient's blood pressure was dynamically monitored and controlled. Indicators such as serum creatinine, the volume of the abdominal drainage, and urine volume were monitored. The severity of complications was evaluated according to Clavien-Dindo classification system (14). Post-operative chemotherapy was continued according to SIOP nephroblastoma guidelines. Abdominal ultrasound, blood urea, serum creatinine, blood pressure, and urine protein content were rechecked one-month post-operatively to determine whether the renal function and tumor recurrence were present. Afterward, the patients underwent follow-up and were reviewed as required.

## Results

### Patient characteristics

From 2018 to 2020, 88 patients diagnosed as WT in our center. Among them, 16 patients were BWT and a total of 8 children with BWT met the inclusion criteria and were enrolled in this study. The average age of onset of the 8 patients was 14 months (7–40 months). Among them, 2 cases were genetically diagnosed as Denys-Drash syndrome, and 2 cases were complicated with hydronephrosis. 37.5% (3//8) of them had multiple tumors. After 2–12 courses of neoadjuvant chemotherapy, all the tumors were stable without significant size reduction. The baseline characteristics of the patients were shown in Table 1.

**Table 1:** The baseline characteristics of enrolled patients.

ID	Gender	Age of onset (month)	Left tumor size (mm)	Right tumor size (mm)	SIOP staging (left/right)	Preoperative chemotherapy cycle	Preoperative Chemotherapy regimen	Response to chemotherapy (RECIST)	Comorbidities
1	Male	12	89 × 65 × 84	82 × 75 × 109, 84 × 74 × 113	II/II	9	ACTD + VCR + DOX, CTX + VP-16	SD	Right hydrocele, left cryptorchidism, hypospadias, DDS syndrome
2	Female	9	84 × 88 × 117	47 × 38 × 57, 22 × 15 × 19	II/II	4	ACT-D + VCR + THP, VCR, VP-16 + CBP, CVD	SD	DDS syndrome
3	Female	7	122 × 96 × 128	47 × 22 × 25	II/II	12	VCR + ACT-D	SD	-
4	Male	40	28 × 24 × 29	69 × 66 × 87	II/III	12	VCR + ACT-D	SD	Lung metastasis
5	Female	34	55 × 56 × 56	93 × 84 × 79	II/III	2	THP + VCR + ACTD	SD	Hydronephrosis, IVC tumor thrombus
6	Female	8	38 × 24 × 34, 17 × 13 × 14	131 × 117 × 155	III/III	7	VAD + VCR	SD	-
7	Female	15	69 × 59 × 45	41 × 27 × 30	III/III	4	CBP + VP-16	SD	-
8	Female	13	83 × 62 × 116	28 × 23 × 26	II/I	6	VCR + ACT-D	SD	Bilateral absence of iris, bilateral cataract, left eye glaucoma

ACTD, actinomycin; VCR, vincristine; DOX, doxorubicin; CTX, cyclophosphamide; VP-16, etoposide; ACT-D, dactinomycin/actinomycin D; THP, pirarubicin; CBP, carboplatin; CVD regimen: cyclophosphamide, vincristine, actinomycin D; VAD regimen: vincristine, doxorubicin, actinomycin D; IVC, inferior vena cava.

### Surgery

Among all the 8 patients, six of them (75%) received staged operation and the other 2 patients (25%) received single-stage operation. In details, 4 patients underwent bilateral autologous kidney transplantation, 2 patients underwent unilateral renal autologous transplantation and contralateral radical nephrectomy, and 2 patients underwent unilateral renal autologous transplantation and contralateral NSS resection. One patient underwent intraoperative inferior vena cava tumor thrombus extraction. Ex vivo tumor resection and kidney autotransplantions were performed on 11 kidneys. Nephron-sparing surgeries were performed on 5 kidneys *in vivo*. The blood loss during unilateral autologous kidney transplantation was less than 50 ml. 2 cases of children undergoing surgery had major intraoperative blood loss and required a blood transfusion to replenish the blood volume. The kidney on the autologous kidney transplant side was reimplanted *in situ*, and the blood flowed smoothly in the blood vessels, without any thrombus or stenosis. Imaging examination of all 8 children, 1 month post-operatively showed no residual tumor. The postoperative pathological diagnosis of all 8 children was nephroblastoma. All of the 11 kidneys who underwent ex vivo tumor dissection and kidney autotransplantation had no residual tumor cell on microscopic surgical margins. Immunohistochemistry (IHC) indicated WT1 positive in 6 patients. Six patients performed IHC tests of Ki67 with varied positive ratio (2%–50%). According to the mid-term results of a stage III randomized clinical trial conducted in our center (ChiCTR-PRRC-12002525), adjuvant radiotherapy combined with chemotherapy didn't show significant efficacy in patients with WT at stage IIIA (15). Thus, all the patients in this study have not received adjuvant radiotherapy.

### Complications

Complications of different severity occurred in 6 children post-operatively, among which, 2 (33.3%) patients had complication of Clavien-Dindo grade IIIa, and 4 (66.7%) patients had complication of grade <3 (Table 2). There were 2 cases of urinary tract infection, 1 case of wound infection, 1 case of adhesive intestinal obstruction, and 4 cases of urinary fistula. All were managed conservatively. 4 patients suffered from postoperative hypertension, and their blood pressure returned to normal after conservative medical therapy. The estimated glomerular filtration rate (eGFR) was 104.38 ± 45.41 ml/min/m^2^ in three days after surgery. Two cases were complicated with Denys-Drash syndrome and therefore suffered from postoperative renal insufficiency. Case 2 had severe proteinuria on admission. After neoadjuvant chemotherapy and staged surgery, her renal function was maintained for 28 months. Then, she underwent allogeneic kidney implantation with both autologous nephrectomies at the same time and kept tumor-free survival for 19 months till now. Case 1 with nephrotic syndrome and WT had a right hydrocele, left cryptorchidism, and hypospadias; consistent with complete Denys-Drash syndrome. He had decreasing eGFR in four months after surgery and then underwent dialysis for a short period (8 months), but died from treatment interruption. So far, 7 children are still tumor-free survival with a median follow-up time of 38 months (range: 24–42 months).

**Table 2:** The perioperative factors of all the enrolled patients.

ID	Surgical stage	Surgical approach	Surgical duration (min) (left/right)	Bleeding amount (ml)	Blood transfusion	Complications (C-D grade)	Post-operative eGFR (ml/min/m^2^)	Post-operative dialysis	WT1(left/right)	Ki-67 rate (left/right)	Survival time (days)
1	Two-stage	Bilateral Kidney Autotransplantation	320/325	20/30	No	Hypertension, renal insufficiency, urinary fistula, urinary tract infection (II)	50	Yes	(−)/(+)	10%/50%	520
2	Two-stage	Left autologous kidney transplantation + Right NSS	220/200	20/20	No	Hypertension, urine leakage (II)	56	No	(−)/(+)	8%/2%	509
3	Two-stage	Left radical resection + Right kidney autotransplantation	265/275	40/35	No	Urinary fistula, hypertension, wound infection (II)	111	No	(−)	NA	617
4	Two-stage	Left kidney autotransplantation + Right kidney radical resection	315/115	30/10	No	No	92	No	(+)	30%	578
5	Single-stage	Left kidney autotransplantation + Right radical resection + IVC tumor thrombus extraction	385	100	No	Urinary fistula, hypertension (IIIa)	89	No	(+)	20%	513
6	Two-stage	Bilateral Kidney Autotransplantation	265/365	150/20	2U	No	188	No	(−)	30%	88
7	Two-stage	Bilateral Kidney Autotransplantation	275/415	50/30	No	Adhesive ileus (I)	86	No	(+)	5%	550
8	Single-stage	Left autologous kidney transplantation + Right NSS	360	100	2U	Pneumonia, urinary tract infection (IIIa)	163	No	NA	NA	403

IVC, inferior vena cava; C-D grade, Clavien Dindo surgical complication grade; eGFR, estimated glomerular filtration rate.

## Discussion

Currently, the diagnosis and treatment strategies for BWT are relatively uniform. They all start with neoadjuvant chemotherapy and then undergo surgical treatment. Intra-operatively, to preserve normal renal function, it is necessary to preserve the nephron as much as possible. Adjuvant chemotherapy should be given according to the highest unilateral stage. The application of neoadjuvant chemotherapy regimens also gives children with BWT the opportunity to undergo NSS (16), thereby avoiding allogeneic kidney transplantation. NSS procedure mainly refers to the resection or stripping of renal tumors. If allogeneic kidney transplantation is performed, it is necessary to both prevent post-transplantation rejection and treat the renal tumors post-operatively. Management is further complicated in patients requiring long-term post-operative hemodialysis. It is therefore essential to intraoperatively preserve the nephron as much as possible. Our study showed that BWT patients with autologous kidney transplantation after extracorporeal tumor resection had 2-year tumor-free survival rate of 87.5% (7/8). Additionally, postoperative complications were all manageable. Taken together, we provided a new treatment strategy to preserve renal units for patients with synchronous BWT.

At present, NSS mainly refers to the resection or stripping of kidney tumors. For small tumors located at the two poles of the kidney, the tumor and surrounding normal kidneys can be resected to ensure negative margins. However, an R0 resection cannot be guaranteed in few cases, namely when the tumor is in the middle of the kidney or close to the collecting system and hilum (17). Performing an NSS procedure in these cases usually results in narrow tumor margin to protect the renal blood supply and avoid damage to the renal hilar vessels. A previous study showed that 31% of children with BWT who underwent NSS surgery, had positive surgical margins and therefore needed further radiotherapy (5).

The bilateral renal tumors in the 8 children with BWT reported in this study were either all quite large, or were located in the middle of the kidney or close to the renal hilum. Performing a traditional NSS would result in a residual tumor, inability to preserve sufficient normal residual renal tissue, and major intra-operative bleeding, which could further affect the judgment of the resection margin, and lead to damage of the renal vessels and pelvis. Intra-operatively, there can also be tumor rupture and dissemination, which increases the risk of postoperative recurrence and metastasis. In contrast, in kidney autotransplantation, resection of both the kidney and the tumor is performed in an isolated bloodless state. In this state, one can easily identify the tumor boundary, resect the tumors completely, reduce marginal tumor residues, retain more nephrons, reduce bleeding, and avoid complications such as high blood pressure and renal ischemia caused by damage to renal blood vessels. Thus, kidney autotransplantation with ex vivo resection of the renal tumor and reconstruction of the organ can be considered the best approach for this kind of complex case and can prevent permanent loss of renal function (18).

Previous reports of the technique mostly focused on benign kidney diseases or adult patients. Up-to-date case reports on pediatric Wilm's tumor were limited. In our study, the duration from kidney resection to HCA cryopreservation solution injection was all within 2 min. This action shortened the renal warm ischemia time, which was significantly shorter when compared to the traditional NSS operation. Perfusion with cryopreservative solution and local cooling of the kidney with ice can preserve more renal function than conventional NSS surgery (19). Furthermore, the kidney was transplanted in the ipsilateral side instead of the contralateral iliac fossa. It is beneficial to use the original arteriovenous position for anastomosis, and maintain the structure and course of the original ureter, which not only simplifies the surgical procedure but also reduces the possibility of malignant tumors spreading to the pelvic cavity.

The most common postoperative complications in the 8 patients included urinary fistula, renal insufficiency, and hypertension. If there is a post-operative long-term urinary fistula, ultrasound-guided renal puncture with catheter drainage should be considered, according to the patient's situation. Perinephric drainage tubes and ureteral stent could be considered during the operation to reduce the morbidity and outcome of urinary fistula. Due to the huge size of the tumor, hyperperfusion nephropathy caused by the remaining normal kidney after removal can result in postoperative hypertension. Relative stenosis caused by intraoperative renal vascular anastomosis may also cause postoperative renal insufficiency or high blood pressure. We suggest paying particular attention to the risk of high blood pressure and using anti-hypertensive drugs early in management. Post-operative urinary tract ultrasonography should be performed regularly to check the patency of renal vessels. This kind of hypertension was often temporary and could be relieved by early diagnosis and treatment.

In this study, 2 children had Denys-Drash syndrome, which is an extremely rare congenital genetic disease. The complete form of Denys-Drash syndrome manifests as diffuse mesangial sclerosing nephrotic syndrome, male pseudohermaphroditism, and Wilms tumor. Incomplete Denys-Drash syndrome has nephrotic syndrome as the main symptom and may be accompanied by male pseudohermaphroditism or Wilms tumor. Treatment with neoadjuvant chemotherapy and surgery should not be delayed even in children with bilateral nephroblastoma who have severe nephrotic syndrome and little normal renal tissue on preoperative evaluation. Such patients should be followed up regularly for the first 2 years after surgery to confirm the absence of tumor recurrence. Subsequently, the need for allogeneic kidney transplantation can be decided based on renal function. Patients with this syndrome may suffer from proteinuria, which can result in a loss of plasma albumin. Meanwhile, residual kidney has the possibility of tumor recurrence. For this situation, we suggest the resection of autologous residual kidney, with simultaneous transplantation of an allogeneic kidney.

The median follow-up time in this study is 38 months and so far, none of the patients had a tumor recurrence. There is a need to further follow up on the long-term efficacy and the recovery of renal function. Furthermore, due to the small sample size, our experience is limited. SIOP-renal tumor study group from Europe and COG from America have conducted several international multi-center clinical trials, which may provide more evidences. In summary, we suggest that if the hospital's surgical technology and perioperative management conditions are feasible, kidney autotransplantation can be a choice for complicated BWT patients with a lower risk of perioperative complications and good prognosis.

## Data Availability

The original contributions presented in the study are included in the article/Supplementary Material, further inquiries can be directed to the corresponding author/s.
